# New Method of Operating for Strabismus by a Temporary Ligature

**Published:** 1853-09

**Authors:** 


*New Method of Operating for Strabismus by a Temporary Ligature.*—M. Tavignot sent in a memoir, the object of which is to explain a new method of operating for strabismus. This new operation is founded on the following idea, that, instead of lengthening a muscle supposed to be too short, you must shorten a muscle in reality too long. ' Instead of leaving the eye to oscillate with difficulty, and sometimes sluggishly, between two muscles, one of which is mutilated by a section, and the other remains always more or less powerless, my method of operating, says the author, attacks the longest muscle, and not only shortens it by a sufficient length to equal that of its antagonist, but it furthermore acts by increasing its physiological contraction.

*First operation.*—The longest muscle—that is to say, that one which is opposed to the deviation being exposed in the ordinary manner for strabotomy, the operator proceeds in the following manner:—A blunt hook, with an eye at its extremity, is passed underneath the muscle, so as by lifting it up to detach it from the globe of the eye. The hook is then carried forward, so that its concavity embraces the muscle at a little distance from its aponeurotic expansion. A thread of silk is then passed though the eye of the hook, then the hook itself is brought towards the operator, leaving the ligature under the muscle. By a double twist of the ends of the thread on one another, a simple, yet very resisting, knot is obtained. There only then remains to finish the operation, to tighten the knot, and cut away one of the ends of the ligature. The other end is brought to the corresponding angle of the eye and fixed to a spot on the circumference of the orbit.

The first effect of this ligature is to render the lateral fibres of the muscles more central, and thus to bring about a shortening of this organ. The second effect is to develop an adhesive inflammation, which not only fixes permanently the abnormal juxtaposition of the muscular fibres, but also establishes adhesion between the muscle and subjacent sclerotic membrane.

The ligature not being intended to produce division of the muscle, must consequently be only temporary. Towards the end of the second, or beginning of the third day, it can be easily taken off by means of gentle traction carefully applied to the end which remains.

This first operation may not in all cases produce the effect which we have described. Very severe strabismus will no doubt prove refractory. It is at least with this idea that I devised a way of making it more efficacious.

*Second operation.*—The hook having been passed under the muscle, as in the preceding case, the ligature is passed, not directly under the muscle, but under the hook, so as to embrace the muscular expansion.

Before going further, it must be discovered by a momentary constriction if the globe is perfectly restored to its normal position. To prove experimentally that the ligature has effected the required degree of shortening, we must proceed, during the operation, in the following manner:—The ligature being passed once under the hook, a different colored thread must be passed through the loop thus formed, then constriction is made by means of the first-mentioned ligature, but taking care to make only one knot, and to make it a single one only. The hook is then withdrawn, and the eye left to itself. The changes in its direction can now be judged of accurately. If the globe is not brought back sufficiently, a larger quantity of muscular tissue must be embraced by the ligature; if the globe is too much brought back, a lesser quantity of muscular tissue must be enclosed; but in either case the ligature already put on must be withdrawn as soon as possible. Owing to the precautions we have adopted with this view, nothing is more easy; the eye being fixed, one end of the ligature is drawn with one hand, while the other hand pulls the thread passed through the loop of this same ligature. The knot gives way immediately to this opposed extension. There only then remains to pass the hook again underneath the muscle (if it has not been already done before taking away the ligature) and recommence the operation, keeping in mind the data furnished by the first trial.—*Dublin Med. Press,* from *Presse Méd. Belge.*

